# Disentangling Hot Carrier Decay and the Nature of Low-*n* to High-*n* Transfer Processes in Quasi-Two-Dimensional Layered Perovskites

**DOI:** 10.1021/acs.jpcc.3c05415

**Published:** 2023-11-27

**Authors:** Lisanne M. Einhaus, Xiao Zhang, Kaijian Zhu, Jeroen P. Korterik, Robert Molenaar, Sven H. C. Askes, Guido Mul, Johan E. ten Elshof, Annemarie Huijser

**Affiliations:** † PhotoCatalytic Synthesis Group, MESA+ Institute for Nanotechnology, University of Twente, 7500 AE, Enschede, The Netherlands; ‡ Inorganic Materials Science Group, MESA+ Institute for Nanotechnology, University of Twente, 7500 AE, Enschede, The Netherlands; § Optical Sciences Group, MESA+ Institute for Nanotechnology, University of Twente, 7500 AE, Enschede, The Netherlands; ∥ NanoBioPhysics Group, MESA+ Institute for Nanotechnology, University of Twente, 7500 AE, Enschede, The Netherlands; ⊥ Department of Physics and Astronomy, Vrije Universiteit Amsterdam, De Boelelaan 1081, 1081 HV Amsterdam, The Netherlands

## Abstract

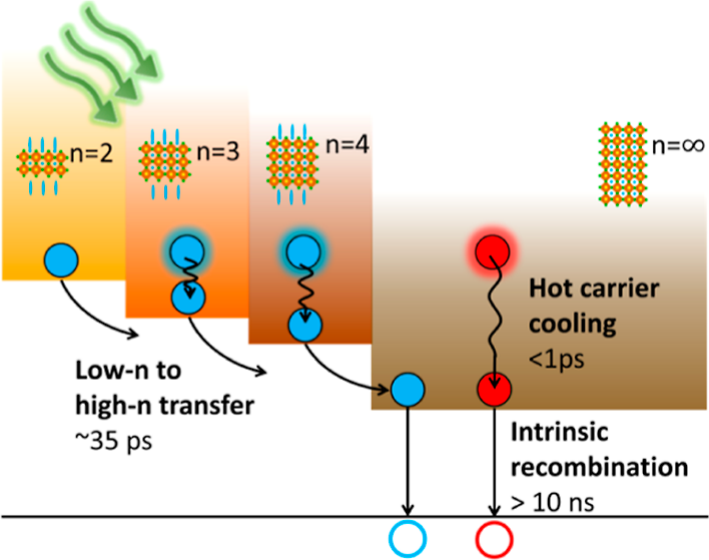

Quasi-two-dimensional (2D) metal halide perovskites (MHPs) are promising photovoltaic (PV) materials because of their impressive optical and optoelectronic properties and improved stability compared to their 3D counterparts. The presence of domains with varying numbers of inorganic layers between the organic spacers (*n*-phases), each with different bandgaps, makes the photoinduced carrier dynamics in films of these materials complex and intriguing. Existing interpretations of the ultrafast femto- or picosecond spectroscopy data have been inconsistent, most of them focusing either on exciton/charge transfer from low-*n* to high-*n* phases or on hot carrier cooling, but not combined. Here, we present a comprehensive study of the carrier dynamics in the Dion–Jacobson type (PDMA)(MA)_(*n*−1)_Pb_*n*_I_(3*n*+1)_ (PDMA = 1,4-phenylenedimethylammonium, MA = methylammonium) perovskite, stoichiometrically prepared as ⟨*n*⟩ = 5. Within the film, a coexistence of various *n*-phases is observed instead of solely the *n* = 5 phase, resulting in an interesting energy landscape for the motion of excitons and charge carriers. We disentangle hot carrier cooling from exciton transfer between low-*n* and high-*n* phases using ultrafast time-resolved photoluminescence and transient absorption spectroscopy. Photophysical modeling by target analysis shows that carrier cooling occurring on a subpicosecond time scale is followed by exciton transfer from low-*n* into high-*n* phases in ca. 35 ps when the film is excited by 532 or 490 nm light. Carriers in the high-*n* phase are much longer lived and decay in a ns time window. Overall, our results provide a comprehensive understanding of the photophysics of this material, which helps to optimize quasi-2D MHP materials for a new generation of PV devices.

## Introduction

Quasi-two-dimensional (quasi-2D) perovskites have recently gained much interest as materials for photovoltaics (PV) and light-emitting diodes because of their improved thermodynamic and environmental stability compared to their well-known three-dimensional (3D) counterparts.^1−7^ In quasi-2D perovskites, inorganic octahedral layers are separated by large organic cations R, thereby forming natural quantum wells. Generally, quasi-2D perovskites can be described by the formula R_*m*_A_*n*–1_B_*n*_X_3*n*+1_ (*m* = 1,2), (*n* = 1,2,3···∞), where A is an organic or inorganic cation (e.g., CH_3_NH_3_^+^ (MA), HC(NH_2_)_2_^+^, and Cs^+^); B is an inorganic cation (e.g., Pb^2+^, Sn^2+^, and Ge^2+^); and X is a halide anion (e.g., I^–^, Br^–^, and Cl^–^). By varying the inorganic layer thickness, it is possible to tune the optical bandgap and the quantum confinement of the material. With decreasing the value of n, the optical bandgap widens and the Coulombic interaction between photoinduced electron–hole pairs increases, leading to bound electron–hole pairs (excitons) instead of free charges.^8,9^

Previous research has established that when a certain stoichiometric mixing ratio ⟨*n*⟩ > 1 is used, multiple microstructural domains with various *n*-values, commonly referred to as *n*-phases, coexist within a film even though the film was intended to be grown as single-phase material.^10,11^ These *n*-phases tend to form a structural gradient, where the low-*n* phases are mostly located close to the substrate at the bottom of the film and the high-*n* phases at the top of the film,^10^ generating an interesting energy landscape for the motion of excitons and charge carriers. Some research groups have managed to fabricate phase-pure ⟨*n*⟩ > 1 films;^12^ however, this requires intricate synthesis methods, e.g., spin-coating dissolved single crystal powders of the desired *n*-value or the use of specific additives.

The most extensively studied type of quasi-2D perovskite is the Ruddlesden–Popper (RP) material. Here, spacer molecules R have one active site that can connect to the inorganic layers. Therefore, a double spacer layer (*m* = 2) is formed in between two adjacent inorganic layers, separated by a van der Waals (VDW) gap.^13^ As an alternative to RP perovskites, Dion–Jacobson (DJ) type quasi-2D perovskites have recently been attracting wide research interest. Contrary to RP type, in DJ type quasi-2D perovskites the spacer molecules have two active sites and can therefore directly bridge two adjacent inorganic layers (*m* = 1). In this way, the distance between two adjacent inorganic layers can be reduced, increasing the interlayer electronic coupling and promoting interlayer charge transport.^13,14^ Because the layers are connected by strong hydrogen bonds instead of weak VDW gaps, the resulting DJ lattice is more rigid, which reduces the electron–phonon coupling and results in a longer carrier lifetime.^15^

In a solar cell configuration, the perovskite layer is typically sandwiched between electron and hole transport layers, each connected to electrodes for efficient charge extraction. To date, RP- and DJ-based perovskite solar cells have reached power conversion efficiencies of 21.07^16^ and 18.82%,^17^ respectively. These records have been achieved by various research strategies, including optimizing quantum well thickness distributions^18^ and improving crystal orientations by vertically aligning the planes.^13^ Further improvements in the solar cell performance require an understanding of the exciton and charge carrier transport mechanisms within the film and their directionality.

The coexistence of the different *n*-phases within a single film results in the presence of multiple bandgaps. The exciton binding energy is high in the low-*n* phases and low in the high-*n* phases, implying excitons in the first and (almost) free electrons and holes in the latter.^19^ Furthermore, excitation above the bandgap results in the formation of hot carriers that could quickly thermalize through coupling to lattice vibrations.^20^ Lead-halide perovskites are known for their relatively slow hot carrier cooling resulting from the hot photon bottleneck.^21^ Analysis of the directionality and dynamics of transfer processes between the different *n*-phases and whether these involve hot or thermalized separate charge carriers or excitons is hence of paramount importance. However, developing mechanistic insight is challenging because of the presence of multiple *n*-phases, the simultaneous presence of excitons and free hot and thermalized carriers, and the occurrence of many different processes in the film, such as hot carrier cooling, exciton, and/or charge transfer processes between the low-*n* and high-*n* phases, charge trapping, and (non)radiative recombination.

A diversity of photophysical interpretations have been published recently. Some studies on quasi-2D perovskite films report exciton (or energy) transfer from low-*n* to high-*n* phases,^22^ while others report electron transfer^23^ in that direction, sometimes in combination with hole transfer in the opposite direction.^10,24,25^ Other studies report a combination of both exciton and charge transfer, occurring at different time scales.^26,27^ The photophysical modeling in many studies is limited, and the interpretation is inconsistent: the focus is either on energy/charge transfer or on hot carrier cooling,^28^ but generally not combined. To the best of our knowledge, only one study by Lin et al. reports subps hot carrier thermalization followed by exciton transport from low-*n* to high-*n* phases in 2–300 ps in RP-type quasi-2D layered perovskites based on PEA with ⟨*n*⟩ = 3 (PEA = C_6_H_5_(CH_2_)_2_NH_3_).^29^ Whether the same mechanism and time scales also apply to other RP and DJ quasi-2D materials is unknown but unlikely since energy levels of the different phases, individual domain sizes, and the lattice rigidity can be expected to play an important role, warranting further studies.

An interesting DJ system is based on the 1,4-phenylenedimethylammonium (PDMA) spacer containing conjugated π-bonds, reducing the exciton binding energy in the 2D phase.^13^ The aromatic ring makes the structure more rigid compared to spacers based on aliphatic chains.^30^ A solar cell based on PDMA with a glass/fluorine-doped tin oxide (FTO)/c-TiO_2_/perovskite/Spiro-OMeTAD/Au device structure achieved an efficiency of 15.81%. The cell was tested in air with 30% relative humidity at room temperature and shows significantly better environmental stability over time than a device based on MAPbI_3_.^31^ The photodynamics of DJ-type quasi-2D lead halide perovskites based on a PDMA spacer (PDMA)(MA)_(*n*−1)_Pb_*n*_I_(3*n*+1)_ (⟨*n*⟩ = 1, 2, 3, 4,···) have not been extensively studied yet, and the findings are contradictory. Zhang et al. studied (PDMA)(MA)_(*n*−1)_Pb_*n*_I_(3*n*+1)_ (⟨*n*⟩ = 4) fabricated by different solution-casting processes by femtosecond transient absorption (TA) using front-side or back-side illumination, indicating carrier transfer from low-*n* to high-*n* quantum wells.^31^ A 1.5 ps component was assigned to ultrafast charge transfer, whereas a 600–900 ps component was attributed to slower carrier transport to the *n* = ∞ phase. However, this study does not elaborate on the photophysical modeling or determination of these values. Yu and colleagues also studied (PDMA)(MA)_(*n*−1)_Pb_*n*_I_(3*n*+1)_ (⟨*n*⟩ = 4) and analyzed the TA data using a 4-component singular value decomposition (SVD).^32^ The 650 fs component was assigned to charge transfer from 2D to quasi-2D phases, the 29 ps component to interfacial charge recombination, the 246 ps component to monomolecular charge recombination, and the >10 ns component to charge carrier recombination. However, no hot carriers were considered, while thermalization can be expected to occur on a subps time scale.^20^ In addition to the ⟨*n*⟩ = 4 material, the same authors also investigated the ⟨*n*⟩ = (6, 8, 10) analogues.^33^ SVD fits at 1 ps were shown; however, the nature of light-induced processes was not discussed. Work by Dučinskas and colleagues excluded energy/charge transfer between low-*n* and high-*n* phases by focusing on a number of ⟨*n*⟩ = 1 materials: (S)PbX_4_, where S = 1,4-phenylenediammonium (PDA), PDMA, or 1,4-phenylenediethylammonium (PDEA), and assigned the positive TA signal to a photoinduced Stark effect.^34^ Qin et al. studied (PDMA)(FA)_*n*−1_Pb_*n*_I_3*n*+1_ layers,^35^ with FA formamidinium, and reported ca. 2 ps Förster type exciton transfer from low-*n* toward adjacent higher-*n* layers.

The present work aims to solve the discrepancy in photophysical interpretations of quasi-2D DJ systems based on a PDMA spacer, (PDMA)(MA)_(*n*−1)_Pb_*n*_I_(3*n*+1)_ (⟨*n*⟩ = 5). We will present a mechanistic photophysical study with the aim to disentangle hot carrier thermalization, the directionality and time scales of transfer processes between low-*n* and high-*n* phases, and whether these involve excitons or charge carriers by combining TA and time-resolved photoluminescence (TRPL) experiments with advanced photophysical modeling by target analysis. Our results provide a comprehensive understanding of the photophysics of this material, which helps optimize quasi-2D metal halide perovskite (MHP) materials for a new generation of PV devices.

## Methods

### Sample Fabrication

The films were made under a dry nitrogen atmosphere by spin-coating perovskite precursor solution on the glass side of indium tin oxide (ITO unpatterned, Ossila)-coated glass. To prepare the (PDMA)(MA)_(*n*−1)_Pb_*n*_I_(3*n*+1)_ (⟨*n*⟩ = 5) layers, precursor solutions were made by dissolving PDMAI_2_, MAI (Sigma-Aldrich, ≥99%, anhydrous), and PbI_2_ (Sigma-Aldrich, 99%) powders in a stoichiometric ratio of 1:4:5 with a Pb^2+^ concentration of 0.6 M in the mixed solvent of *N*,*N*-dimethylformamide (DMF, Sigma-Aldrich, 99.8%) and dimethyl sulfoxide (DMSO, Sigma-Aldrich, ≥99.9%) at a ratio of 10:1. The PDMAI_2_ powder was synthesized in-house by first dissolving PDMA [*p*-xylylenediamine, Sigma-Aldrich, 99%] in EtOH while stirring the solution. Then a stoichiometric amount of HI [hydroiodic acid 57%, EMSURE] was added with a molarity double that of PDMAI_2_. The solution was heated in an oil bath at 100 °C until all liquid was evaporated and then purified with diethyl ether (Sigma-Aldrich, ≥99.9%) until it got clear and nearly colorless. The precipitates were then dried in a vacuum oven at 60 °C for a few days. To produce the thin films, the hot-casting method was employed. The ITO-covered glass substrates were first cleaned and treated with O_2_-plasma and then preheated on a 100 °C hot plate for 10–15 min. The precursor solution was subsequently spin-coated onto the hot substrate with a stepwise program of 1500 rpm for 15 s, followed by 4000 rpm for 20 s. Finally, the resulting film was formed by postannealing on a 100 °C hot plate for 10 min.

### UV–Vis Spectroscopy

Transmittance spectra were recorded by using a PerkinElmer Lambda 950 UV–vis spectrometer equipped with an integrating sphere. During the measurements, the samples were contained in a quartz cuvette filled with nitrogen to prevent contact with moisture and oxygen.

### Steady-State PL

Steady-state PL measurements were performed using a homemade setup. A 405 nm continuous wave laser source (MatchBox series, Integrated Optics) was connected by a fiber to a black 3D-printed measurement chamber, where the light was directed perpendicularly onto one of the samples. Light emitted by the sample was collected by another fiber connected to a BLUE-Wave VIS-200 Spectrometer, while residual laser light was filtered out by a 435 nm long-pass filter. The excitation intensity used ranged from 11 to 25 mW.

### Time-Resolved Photoluminescence

Fluorescence spectra and lifetimes with high time resolution were recorded with a Hamamatsu streak camera (C10910) equipped with a synchroscan sweep module (M10911–01). The samples remained in a nitrogen-filled cuvette throughout the process, from fabrication to measurement. The samples were excited using pulses with a center wavelength of 532 nm from a Fianium femtosecond laser with a pulse duration of 300 fs full width at half-maximum (FWHM) at a repetition rate of 80.37 MHz. The intensity of the excitation beam was kept below 5 mW at the sample position to avoid photobleaching of the sample. A quartz lens with a 50 mm focal length was used to focus the laser beam onto the sample, which was contained in a quartz cuvette filled with nitrogen. The emitted light was collected and focused on the input of the spectrograph (Acton SP2300, Princeton Instruments, using the grating with 50 lines/mm blazed at 600 nm) by two 2 in. diameter, 50 mm focal length glass lenses. A 570 nm long-pass filter was placed in front of the spectrograph to block the scattered laser light and protect the device from potential damage. The output of the spectrograph was directed to the photocathode of the streak camera. To correct for the spectral sensitivity of the setup, a spectral sensitivity correction was performed based on the measured and provided emission spectrum of a blackbody calibration lamp (Ocean Optics, HL-2000).

### Time-Correlated Single Photon Counting

To measure PL lifetimes beyond the 1–2 ns accessible by the streak camera, a time-correlated single photon counting (TCSPC) laser scanning confocal microscope (PicoQuant, MT200) was used. The samples remained in a nitrogen-filled cuvette from fabrication in the glovebox to the microscopy studies. The samples were excited using a pulsed laser source at a repetition rate of 2 MHz (PicoQuant, LDH-D-C-485) with an excitation wavelength of 485 nm and a pulse duration of ∼100 ps FWHM. The laser light was directed toward the sample using a dichroic mirror (Chroma, ZT405/488rpc-UF3). The sample was illuminated by a 20× objective (Olympus, LUCPlanFL N 20×) with a power density of approximately 1 W/cm^2^, and the PL was collected by the objective and directed through a pinhole toward three single photon avalanche detectors (SPAD, Excelitas, SPCM-AQRH-14-TR). Each SPAD was set to a specific spectral region: green 520/35 nm band-pass, orange 620/60 nm band-pass, and red 650 nm long-pass. Further lifetime analysis was based on the red channel, which exhibited the most significant intensity. The lifetime histograms were processed using a custom Python script that fits the histograms to a third-order exponential model using a nonlinear least-squares minimization method. The script then calculated the intensity-weighted average lifetime from the fitted model.

### TA Spectroscopy

The femtosecond TA spectroscopy (fs TA) system included a Coherent Micra seed laser that generated 800 nm pulses with a pulse duration of 35 ± 1 fs (FWHM) at an 80 MHz repetition rate. These pulses were amplified to 800 nm pulses at 5 kHz repetition rate by using a Coherent Legend Ti:sapphire amplifier. The output was split into pump and probe beams with an 85:15 beamsplitter. The pump beam was passed through an optical parametric amplifier system (TOPAS—Prime with NirUVis extension, Light Conversion) to generate 490 nm pulses, and chopped to 2.5 kHz to provide a “pump on” and “pump off” mode to determine the differential absorbance. The probe beam was guided through a mechanical delay stage and subsequently focused into a moving CaF_2_ crystal (Newlight Photonics, 3 mm thickness) to generate a white light continuum. The crystal was mounted on a motorized translational stage and moved at ca. 2 mm/s to avoid thermal damage. The remaining 800 nm of light was filtered out of this beam by using a short-pass filter. An angle of 54.7° (magic angle) was set for the polarization of the probe beam relative to the pump beam polarization, to avoid anisotropy effects.^36^ The pump and probe beams were focused to overlap on the sample, with spots of approximately 250 and 50 μm diameter, respectively. The transmitted pump beam was blocked, and the transmitted probe beam was directed toward a home-built detector system consisting of a 15 cm spectrograph and a 256 pixels diode array detector. The samples were placed in a quartz cuvette with the lid sealed with parafilm and were never in contact with oxygen or moisture, still within the nitrogen environment of the glovebox. We observed earlier that such sealing excludes any O_2_-induced reduction in phosphorescence lifetime of Ru–polypyridyl complexes dissolved in acetonitrile and purged with N_2_ for at least 1 day.^36^ During the measurement, the samples were mounted on a continuously moving translational stage to prevent photobleaching of the measurement area, and the transient signal was verified to remain similar during the experiment. As our focus is on ultrafast processes, the early time delay steps were set to small values (20 fs up until 2.5 ps and 30 fs up until 5 ps) and gradually increased with delay time. Although the continuum is stable, strong light absorption by the sample implies that fewer probe photons are able to reach the detector, lowering the signal-to-noise ratio. As a result, the signal-to-noise ratio depends on the probe wavelength and is the best in the case of weak absorption by the sample. This does not allow TA measurements at shorter wavelengths, as the absorbance of our samples is very high in that wavelength region. In the case of the TA measurement at 490 nm excitation, a few more photons in the short wavelength range could reach the detector due to a slightly thinner sample, enabling a broader wavelength region to be measured. We decided to not fabricate thinner layers, as this would lower the TA and PL signals and therefore require higher pump intensities, leading to photobleaching and second-order photophysical processes and also influence the crystallization and the distribution of *n*-phases. The obtained TA spectra were analyzed using target analysis in the open-source program Glotaran.^37^ The pump power was kept relatively low (4–12 μW) and verified to be in the linear regime, where the intensity of the transient signal increased linearly with the incident pump intensity.

## Results and Discussion

### Steady-State Characterization

**Figure 1:**
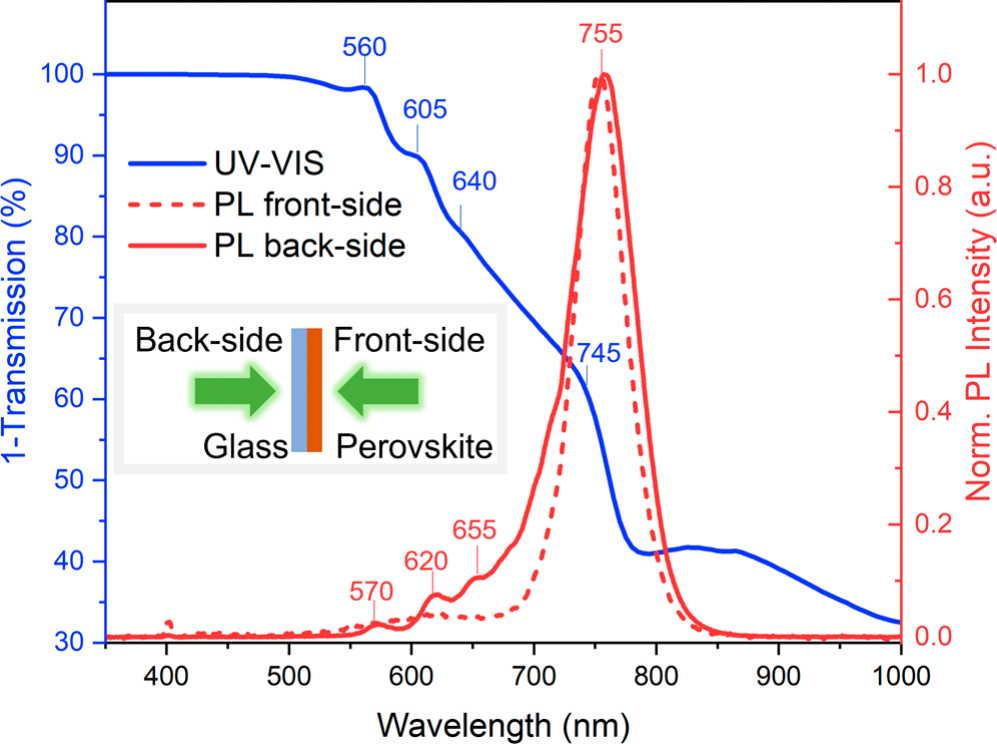
Steady-state UV–vis absorption and PL spectra of (PDMA)(MA)_(*n*−1)_Pb_*n*_I_(3*n*+1)_ (⟨*n*⟩ = 5).

Figure 1 shows the steady-state UV–vis absorption and PL spectra of the as-prepared (PDMA)(MA)_(*n*−1)_Pb_*n*_I_(3*n*+1)_ (⟨*n*⟩ = 5) films. At ca. 745 nm, an absorption onset is present typical for the 3D phase (*n* = ∞), while excitonic absorption bands are observed at around 560 nm (*n* = 2), 605 nm (*n* = 3), and 640 nm (*n* = 4). The signal in the (near-)IR is due to reflection of the sample.

The PL spectrum shows a significant dependence on the direction of illumination, i.e., front-side or back-side of the sample. In both cases, a PL band around 755 nm is detected, originating from high-*n* phases. Illumination from the back-side shows multiple narrow PL bands, most distinct at 570, 620, and 655 nm, which we assign to PL from multiple low-*n* phases. These bands are not observed under front-side illumination, indicating that the low-*n* phases are mostly located at the back-side of the sample. Illumination from the front-side shows a very broad and featureless weak PL band centered around 620 nm, which may originate from semiamorphous mixed-*n* domains. Consistent with the work of Dučinskas,^31^ no *n* = 1 UV–vis absorption band centered around 508 nm or PL band around 518 nm are resolved for this layer.^34^ The steady-state absorption and PL spectra indicate that the fabricated films indeed contain a structural gradient, with the low-*n* phases mostly located close to the substrate at the bottom of the film and the high-*n* phases at the top of the film. These films are hence suitable for investigating the photophysical properties and the role of the structural gradient in the directionality and ultrafast dynamics of the transfer processes of hot and thermalized charge carriers or excitons between the individual *n*-phases.

### Time-Resolved Photoluminescence

**Figure 2:**
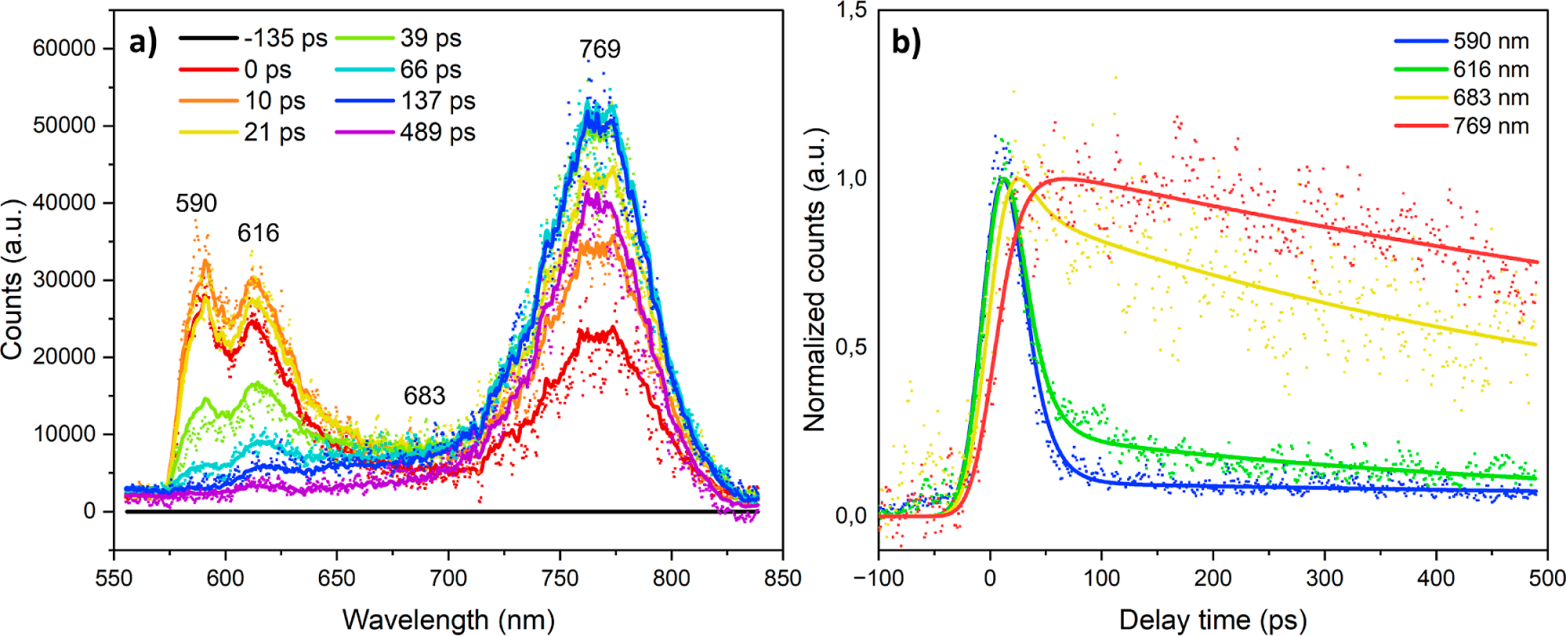
TRPL spectra at various times after 532 nm excitation (a) and kinetic traces normalized to 1 at selected PL wavelengths (b) measured by streak camera detection of (PDMA)(MA)_(*n*−1)_Pb_*n*_I_(3*n*+1)_ (⟨*n*⟩ = 5) recorded using back-side illumination and measured in reflection mode. Note that the spectrum is cutoff by a long-pass 570 nm filter.

To study the potential occurrence of exciton transfer and the dynamics involved between the low-*n* and high-*n* phases and electron–hole recombination over time, TRPL measurements were conducted. These measurements were performed in reflection mode, with the back-side of the samples illuminated. The TRPL spectra (Figure 2a) recorded at various times after photoexcitation at 532 nm show three main PL features, centered around 590, 616, and 769 nm, analogous to the steady-state PL spectrum shown in Figure 1, with the small deviations likely due to the different experimental setups used. In between these main bands, a very broad and featureless PL band is observed, as indicated in Figure 2a at 683 nm.

Note that the spectrum is cutoff by the 570 nm long-pass filter, thereby not fully resolving the PL band at the highest energy, which is expected to have a maximum around 570 nm according to the steady-state PL spectra (Figure 1). The PL bands observed around 590 and 616 nm develop within the experimental time resolution. Very interestingly, the dynamics of the PL band around 769 nm differ significantly from those at 590 and 616 nm, with the latter two already decaying while the intensity of the first is still increasing. This difference is highlighted by the kinetic traces shown in Figure 2b, indicating that the low-*n* PL bands around 590 and 616 nm fully develop within the instrumental response time following excitation and subsequently decay very quickly. Conversely, the high-*n* PL band around 769 nm increases more slowly following photoexcitation, with a rise that seems correlated to the decay of the signal of the low-*n* phases and exhibits a much slower decay. The broad, featureless band around 683 nm seems to show a combination of both kinetics. These dynamics indicate the occurrence of exciton transfer from the low-*n* to high-*n* phases, with possibly also a contribution from emission reabsorption.^38^ Upon front-side illumination, only the high-*n*-phase PL band is resolved (Figure S4). Illumination from this side also shows a relatively slow rise of the high-*n* signal, analogous to back-side illumination, albeit slightly faster (Figure S5). This is likely due to more excitation of the high-*n* relative to the low-*n* phase, and confirms the occurrence of energy transfer from the low-*n* to the high-*n* phase. In order to determine the rates for energy transfer from low-*n* to high-*n* and the radiative decay of charge carriers in the latter, the TRPL data were modeled using target analysis.^37^ The photophysical model is based on 3 components: the low-*n* phase, the high-*n* phase, and the semiamorphous mixed *n*-phase, giving rise to the very broad and featureless PL discussed above. All 3 components are assumed to be formed within the instrument response time (IRT) of the streak camera setup. Hot carriers initially generated are presumed to have already been cooled to the band edge within the IRT.^29,39,40^ In our model, the low-*n* phase decays with the decay rate *k*_1_ into the high-*n* phase, which then intrinsically decays with *k*_3_. The mixed phase independently decays with *k*_2_. This model describes the TRPL data well, as is clear from the fits included as solid lines in Figure 2. The resulting time constants are presented in Table 1, and the obtained species-associated spectra are presented in Figure S3 (Supporting Information). The low-*n* phase exhibits the shortest lifetime (∼16 ps), approximately similar to the IRT of the streak camera for the used time range and slit widths, and a more accurate value is obtained by femtosecond TA experiments as discussed below. The mixed *n*-phase shows a longer lifetime (445 ± 7.82 ps), while the estimated high-*n* lifetime is even longer (∼1.59 ± 0.01 ns) and only partially decays in the experimental time window of the streak camera setup.

**Table 1:** Time Constants Obtained from Target Analysis of the TRPL Data Based on the 3-Component Model Described in the Main Text

IRT = 16.00 (ps)	low-*n*	mixed *n*-phase	high-*n*
decay rates (ps^–1^)	*k*_1_ = 0.0640 (±0.936)^a^	*k*_2_ = 0.00224 (±3.86 × 10^–5^)	*k*_3_ = 0.000627 (±2.75 × 10^–6^)
lifetimes (ps)	τ_1_ = ∼16^a^	τ_2_ = 445 (±7.82)	τ_3_ = 1.59 (±0.01) × 10^3^

a A more accurate value is obtained from fs TA, as discussed below.

**Figure 3:**
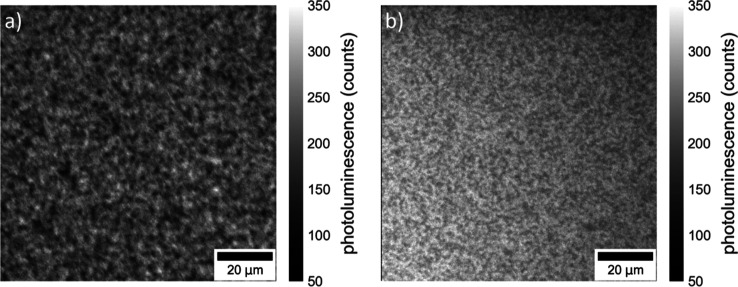
Confocal PL intensity maps of (PDMA)(MA)_(*n*−1)_Pb_*n*_I_(3*n*+1)_ (⟨*n*⟩ = 5) at wavelengths >647 nm either excited from the back-side (a) or the front-side (b) of the sample recorded using 485 nm excitation.

To better quantify the emissive decay of the high-*n* phase and exclude potential effects from the back sweep of the streak camera on lifetimes >1 ns, time-resolved PL measurements using TCSPC confocal microscope detection with a time window of ∼500 ns using 485 nm pulsed excitation at 2 MHz repetition rate were performed. Also, potential charge accumulation effects will be insignificant at this lower repetition rate, likewise in the femtosecond TA experiments discussed below. Overview images were made by measuring the PL in the wavelength range above 647 nm using back-side or front-side illumination (Figure 3). These images indicate the homogeneity of the sample. In both images, the emitting material appears to be homogeneously distributed over the sample.

**Figure 4:**
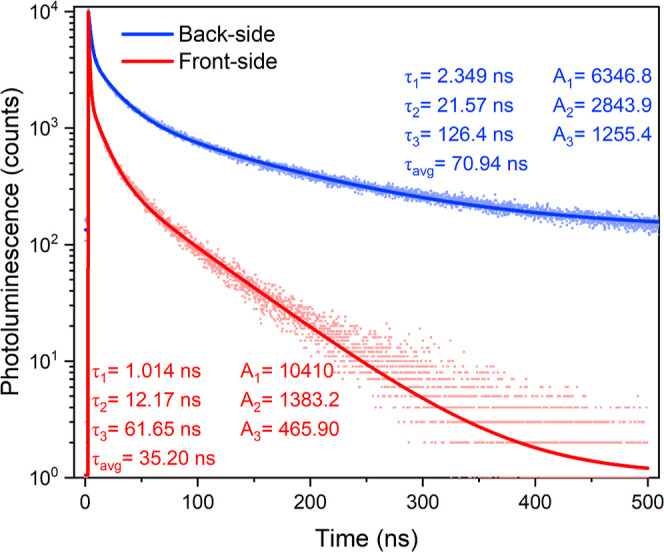
Typical PL decay of (PDMA)(MA)_(*n*−1)_Pb_*n*_I_(3*n*+1)_ (⟨*n*⟩ = 5) measured by TCSPC detection at PL wavelengths >647 nm obtained by exciting at 485 nm either from the front-side or the back-side of the sample. The solid lines indicate fits with a third-order exponential; the obtained fit parameters are shown in the inset.

To accurately determine the PL lifetimes, point measurements were performed at 16 locations distributed in a 4 × 4 grid over the sample area. Typical PL lifetime traces for back-side and front-side illumination at 485 nm are shown in Figure 4. The data were fitted with a custom-made Python script using third order exponential fits. As the measured decay is substantially slower than the IRT, the IRT was set to be infinitely fast to improve the quality of the parameters obtained from fitting. Average lifetimes τ_avg_ were obtained using the following formula: τ_avg_ = (*A*_1_ × τ_1_^2^ + *A*_2_ × τ_2_^2^ + *A*_3_ × τ_3_^2^)/# counts, where *A*_*n*_ indicates the amplitude of the *n*th component, τ_*n*_ is the lifetime of the *n*th component, and # counts is the total number of measured photons on which the fit is based. When the back-side of the sample is illuminated, the average time delay τ_avg_ is measured to be 70.89 ± 1.53 ns. On the other hand, when the front-side of the object is illuminated, the average time delay τ_avg_ is measured to be 34.89 ± 0.98 ns. These values were obtained by averaging over the respective 16 data sets.

### Transient Absorption

In order to obtain a better understanding of the exciton and charge carrier dynamics within the material and, in particular, characterize and disentangle the fast exciton transfer indicated by TRPL and hot carrier cooling, femtosecond TA measurements were performed. The sample was photoexcited from the back-side to excite as much of the low-*n* phase as possible such that the exciton transfer process between the different phases could be investigated. Moreover, back-side illumination does not give the very broad and featureless weak PL band around 620 nm, potentially originating from semiamorphous mixed-n domains. To disentangle the decay of hot carriers within the individual low-*n* and high-*n* phases vs exciton transfer between the *n*-phases, experiments were performed using three different photoexcitation center wavelengths: 630, 532, and 490 nm.

**Figure 5:**
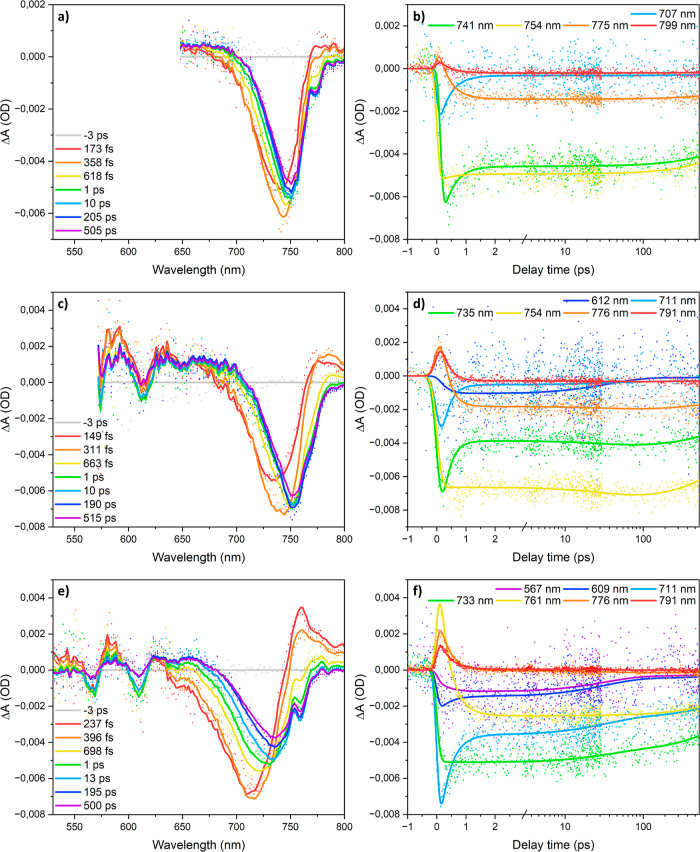
TA spectra of (PDMA)(MA)_(*n*−1)_Pb_*n*_I_(3*n*+1)_ (⟨*n*⟩ = 5) at the indicated delay times under (a) 630, (c) 532, and (e) 490 nm excitation from the back-side of the film. The corresponding kinetic traces at key probe wavelengths are shown in (b,d,f).

At 630 nm excitation, the photon energy is too low to excite the *n* = 1 and *n* = 2 phases. However, it is sufficient to excite the higher-*n* phases and uncover their photodynamics. The corresponding TA spectra at various time delays following photoexcitation are shown in Figure 5a. At early times (<1 ps), the spectra show a negative signal around 740 nm, corresponding to ground state bleaching (GSB) of the high-*n* phase. The GSB red-shifts within ca. 1 ps toward 750 nm. Analogous to earlier work,^27,41^ we assign this ∼10 nm red shift with time to many-body interactions and hot carrier cooling toward the band-edge. The hot carriers also give rise to a small photoinduced absorption (PIA) band centered around 770 nm, as also observed previously in films of similar composition ((PDMA)(MA)_(*n*−1)_Pb_*n*_I_(3*n*+1)_ (⟨*n*⟩ = 4)),^31^ as well as in other low-dimensional perovskite films^42^ or films of the 3D perovskite MAPbI_3_.^43^ The intensity of this band is weak compared to the excitation at 532 or 490 nm discussed below, as expected. Furthermore, a very weak positive PIA <700 nm overlapping with the GSB is observed, which can be assigned to absorption by thermalized carriers in the high-*n* phase.^44^ Possible explanations for this feature include a shifted continuum^45^ or a photoinduced change in refractive index.^46^ Notably, in the 770–820 nm wavelength range, some oscillations are visible in the TA spectra. This is likely due to interference caused by the interface between the sample substrate and the cuvette in which the sample is kept. Decay of the transient signal due to charge carrier recombination barely occurs in the subns TA time window, which agrees with the long-lived PL of the high-*n* phase (Figure 3).

The second excitation wavelength used was 532 nm, similar to the excitation wavelength used in the TRPL measurements, enabling a relatively straightforward comparison between the two sets of data. Excitation of the *n* = 1 phase is still not favored under these conditions. However, the other low-*n* phases are excited, and hot carriers are generated. The TA spectra are shown in Figure 5c. Similar to the 630 nm pump, a high-*n* GSB is observed, which now red-shifts within ca. 1 ps from 730 to 753 nm. This red shift is more pronounced due to the fact that the photon energy of 532 nm light exceeds the bandgap more than the photon energy of 630 nm light, which is favorable for the generation of hot carriers. As a result, the PIA around 770 nm at early times is also more intense. Similar to excitation at 630 nm, a very broad PIA <700 nm is observed, which is now overlapping with two negative features around 614 and 650 nm, which correspond to the GSB of the *n* = 3 and *n* = 4 electronic transitions, respectively. The onset of a *n* = 2 GSB around 570 nm is just visible, though at the edge of our spectral measurement window due to the high absorption of our samples <570 nm.

The final excitation wavelength used was 490 nm, with a photon energy above the absorption edges of all individual phases. The obtained TA spectra using this pump wavelength are shown in Figure 5e. Similar to the other pump wavelengths, the TA spectra show the negative GSB signal of the high-*n* phase and the PIA <700 nm. The red shift of the high-*n* GSB with time is even more pronounced compared to excitation at 630 or 532 nm, likely as a result of the high photon energy used, with the GSB shifting from 710 to 740 nm. In addition, the PIA around 770 nm is also more intense, as anticipated since the higher photon energy with 490 nm excitation compared to especially 630 nm or to a lower extend to 532 nm is favorable for the generation of hot carriers, even in the low-*n* phases. As expected, the TA spectra again show negative features centered around 565, 610, and 645 nm, corresponding to GSB of the *n* = 2, 3, and 4 electronic transitions, respectively. Note that these values are blue-shifted by ca. 5 nm relative to those at 532 nm excitation, which may be due to less excitation of high-*n* phases and therefore less spectrally overlapping PIA <700 nm assigned to absorption by thermalized carriers in the high-*n* phase.^44^

Figure 5b shows the kinetic traces at selected wavelengths for the 630 nm excitation. The negative signal at 707 nm fully develops within the TA IRT (∼100–150 fs) and subsequently decays within 1 ps, indicative of hot carrier cooling. The negative signal at 741 nm seems to develop slightly slower than the IRT, shows a partial ∼1 ps decay, and then remains constant within the experimental time window. Figure 5d,f presents the kinetic traces at selected wavelengths for 532 and 490 nm excitation, clearly showing the ca. 1 ps red-shift in high-*n* GSB due to hot carrier cooling and the associated decay in PIA >770 nm. The GSB signals of the *n* = 2, *n* = 3, and *n* = 4 phases are also resolved and spectrally overlap with a weak PIA <700 nm assigned to thermalized carriers in the high-*n* phase.^44^ Note that the strong absorption of our samples at the lower wavelengths decreases the signal-to-noise ratio in this spectral range. For *n* = 2 and *n* = 3, the GSB signals decay in a few tens of picoseconds, likely demonstrating the low-*n* to high-*n* exciton transfer process causing the fast decay of the low-*n* PL discussed above. Notably, the high-*n* GSB for 490 nm excitation shows slightly stronger decay on a subnanosecond time scale compared to 630 and 532 nm excitation. Such decay is also observed in the previously discussed TRPL data (Figure 2) recorded using 532 nm excitation. We tentatively assign this small difference to the structural inhomogeneity of the high-*n* phase domains, which may also explain the brighter and darker PL areas (Figure 3). For 490 nm excitation, we also observe that during the recording of multiple TA data sets, the lifetime of the high-*n* phase signal becomes shorter over time. The same effect was also observed during the TCSPC measurements and is possibly caused by photodoping.^47^

### Photophysical Modeling

**Figure 6:**
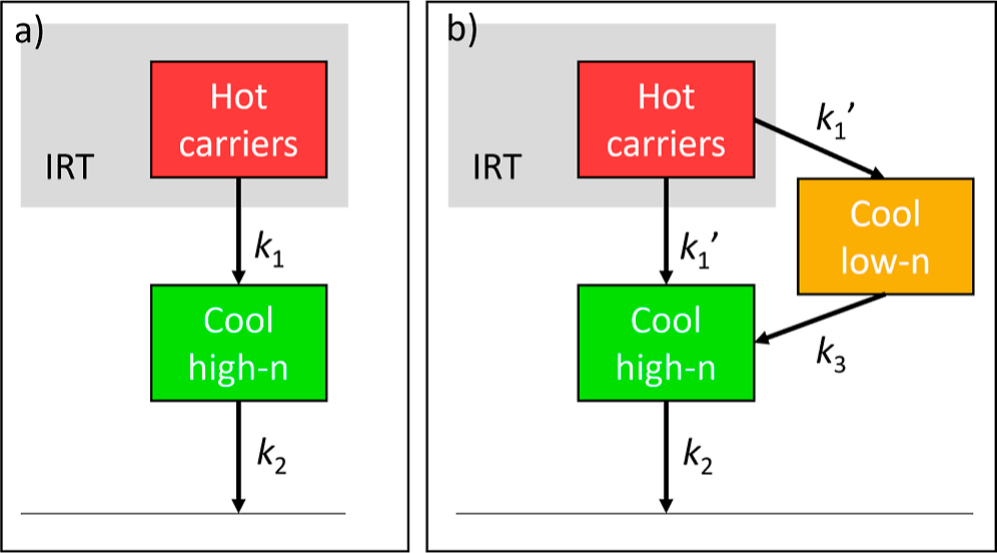
Photophysical models used for target analysis of (PDMA)(MA)_(*n*−1)_Pb_*n*_I_(3*n*+1)_ (⟨*n*⟩ = 5) under (a) 630 and (b) 532 and 490 nm excitation. IRT = instrumental response time.

In order to analyze the photophysical processes discussed earlier in a quantitative manner, photophysical modeling by target analysis was conducted on the TA data using the photophysical models shown in Figure 6. To prevent overmodeling, we aimed to model the data using the fewest parameters while still providing a good fit and rational species-associated spectra. For excitation at 630 nm, the model consists of two species: hot carriers and thermalized high-*n* carriers, as shown in Figure 6a. Though according to the UV–vis absorption spectrum (Figure 1) also the *n* = 4 phase can be excited, its contribution to the TA spectra is negligible, possibly due to a weak absorption relative to the high-*n* phase. According to this model, hot carriers are generated within the IRT by the pump pulse since the photon energy is above the bandgap of the high-*n* phases in the film. These hot carriers then thermalize with decay rate *k*_1_ into thermalized high-*n* species, which further decay to the ground state with decay rate *k*_2_.

For excitation at 532 and 490 nm, a low-*n* phase component is added to the model, as shown in Figure 6b. In this case, hot carriers are formed within the IRT in both the high-*n* and low-*n* phases, which can thermalize with decay rate *k*_1_′ into thermalized charge carriers in the high-*n* phase and thermalized excitons in the low-*n* phase. Carrier temperatures were extracted for excitation at 490 nm by fitting the high-energy side of the high-*n* GSB with a Maxwell–Boltzmann distribution (see “Extracting Tc from TA data”, Supporting Information). Note that the PIA band centered around 770 nm does not necessarily indicate hot carriers in all low-*n* phases, including *n* = 1. To connect the two models, we assume *k*_1_ = 2 × *k*_1_′ for excitation at 532 or 490 nm, as there are two parallel hot carrier decay pathways instead of just one as for 630 nm excitation. The excitons in the low-*n* phases can subsequently undergo energy transfer to the high-*n* phase with a rate of *k*_3_. This process is assumed to outcompete intrinsic (non)radiative decay in the low-*n* phases because the fast PL decay of the low-*n* phases is correlated to the rise of the PL of the high-*n* phase (Figure 2b). Although the approximately 5 nm blue-shift of the TA spectra for 490 nm excitation might indicate the transfer of only part of the excitons from low-*n* to high-*n* phases, intrinsic low-*n* decay has not been included in the model to prevent overmodeling, as including this would not yield unique rate constants. Finally, the charge carriers in the high-*n* phase decay to the ground state with *k*_2_. The TA fits resulting from these models are included as solid lines in Figure 5. Although these models are likely a simplification of reality, they describe the TA data well. The obtained time constants are presented in Table 2. The obtained ultrafast hot carrier thermalization rate into thermalized high-*n* carriers ranges from ca. 2.53 ps^–1^ (*k*_1_, 630 nm excitation) to ca. 1.36 ps^–1^ (*k*_1_′, 490 nm excitation), consistent with values of <1 ps reported in literature.^29,39,40^ The transfer of excitons from the low-*n* phase to the high-*n* phase (*k*_3_) subsequently occurs in ca. 0.03 ps^–1^, which is in the same range that analyzed using TRPL (Table 1). Finally, the intrinsic decay of the high-*n* phase (*k*_2_) occurs at 2.1 × 10^–4^ to 4.2 × 10^–4^ ps^–1^. The decay of charge carriers in the high-*n* phase is not fully resolved in TA due to the limited time window, though from the TCSPC measurements we know this contains components of ca. 2.3, 22, and 126 ns, consistent with literature reporting >10 ns lifetimes.^32^

**Table 2:** Time Constants Obtained from Target Analysis of the TA Data for 630, 532, and 490 nm Excitation

	630 nm excitation	532 nm excitation	490 nm excitation
Process, Rate Constant (ps^–1^)			
hot carrier cooling, *k*_1_ or *k*_1_***	2.53 (±0.081)	1.91 (±0.12)	1.36 (±0.028)
high-*n* intrinsic, *k*_2_	2.10 × 10^–4^ (±1.2 × 10^–5^)	3.22 × 10^–4^ (±3.3 × 10^–6^)	4.21 × 10^–4^ (±2.7 × 10^–5^)
low-*n* to high-*n*, *k*_3_		0.0273 (±2.1 × 10^–3^)	0.0303 (±1.7 × 10^–3^)
Species, Lifetimes (ps)			
hot carriers, τ_1_	395 × 10^–3^ (±0.013)	261 × 10^–3^ (±7.8 × 10^–3^)	367 × 10^–3^ (±0.017)
high-*n*, τ_2_	4.76 × 10^3^ (±2.9 × 10^2^)	3.11 × 10^3^ (±3.6 × 10^2^)	2.38 × 10^3^ (±1.7 × 10^2^)
low-*n*, τ_3_		36.6 (±3.0)	33.0 (±2.0)

**Figure 7:**
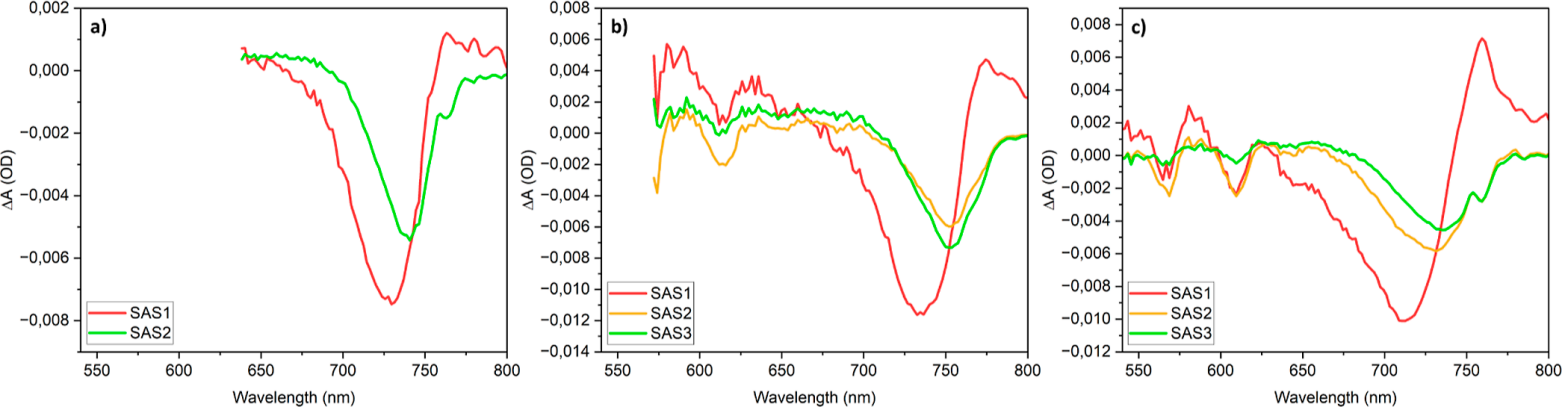
Species associated spectra obtained from target analysis of the TA data of (PDMA)(MA)_(*n*−1)_Pb_*n*_I_(3*n*+1)_ (⟨*n*⟩ = 5) for photoexcitation at (a) 630 nm, (b) 532 nm, and (c) 490 nm.

Figure 7 presents the species-associated spectra obtained from target analysis for 630, 532, and 490 nm. The red lines represent the TA spectra of the hot carriers, showing all negative GSB surrounded by positive PIA. The green lines represent the TA spectra of thermalized high-*n* phase carriers, as their main features are GSB and PIA associated with the high-*n* phase. These spectra show a negative GSB around 740 nm for excitation at 630 nm, around 757 nm for excitation at 532 nm, and around 736 nm for excitation at 490 nm, accompanied by a broad positive signal at the high-energy side, as is typical for the 3D phase.^44^ The orange lines represent the TA spectra of thermalized low-*n* excitons, as these spectra feature various low-*n* GSB. These spectra exhibit negative GSB features around 567, 610 (615 nm), and 645 nm (650 nm), corresponding to the *n* = 2, 3, and 4 phases when excited at 490 nm (532 nm), overlapping with a weak PIA which can be assigned to absorption by thermalized carriers in the high-*n* phase.^44^

The present study is the first to disentangle hot carrier thermalization in quasi-2D materials in either the low-*n* phases or the high-*n* phases from exciton or charge transfer processes from the first to the latter and to resolve their nature. We combined time-resolved fluorescence and femtosecond TA spectroscopy with target analysis of the spectrotemporal response and included the photoexcitation photon energy as a parameter in the photophysical modeling. Hot carriers initially generated by photoexcitation quickly (subpicoseconds) decay into thermalized carriers either in the low-*n* or high-*n* phases. This process is followed by exciton transfer from the low-*n* to the high-*n* phases in ca. 35 ps. Charge carriers in the high-*n* phases are long-lived, with up to tens of ns lifetimes. In summary, our results provide a comprehensive understanding of the photophysics of this material, which helps in designing quasi-2D MHP for a new generation of PV devices.

## Conclusions

This study investigates the carrier dynamics in the quasi-2D layered perovskite (PDMA)(MA)_(*n*−1)_Pb_*n*_I_(3*n*+1)_ (⟨*n*⟩ = 5) using various ultrafast spectroscopic techniques, including TRPL, TCSPC, and TA. The perovskite film consists of different quasi-2D perovskite phases, with the low-*n* phases primarily on the substrate side and high-*n* phases on the nitrogen side. Hot carrier cooling dynamics in the individual phases are disentangled from transfer processes between the low-*n* and high-*n* phases, and the latter is observed to occur via exciton transfer from the low-*n* to the high-*n* phase and possibly also some emission reabsorption. Carrier cooling occurs on a subpicosecond time scale, with the subsequent energy transfer from low-*n* to high-*n* phases occurring in ca. 35 ps. Carriers in the high-*n* phase are much longer-lived and decay in a ns time window. The present study offers unique mechanistic insights essential for the optimization of quasi-2D perovskite materials for a new generation of PV devices.

## Supporting Information Available

Disentangling hot carrier decay and the nature of low-*n* to high-*n* transfer processes in quasi-two-dimensional layered perovskites; XRD pattern of (PDMA)(MA)_(*n*−1)_Pb_*n*_I_(3*n*+1)_ (⟨*n*⟩ = 5) perovskite film; SEM images of (PDMA)(MA)_(*n*−1)_Pb_*n*_I_(3*n*+1)_ (⟨*n*⟩ = 5) perovskite thin film, cross-sectional and plan view; species-associated spectra obtained from target analysis on TRPL data for photoexcitation at 532 nm; TRPL spectra and kinetic traces under front-side photoexcitation at 532 nm; and procedure and results related to the extraction of carrier temperatures from the TA data (PDF)
