# Supplementary material for: Disentangling Hot Carrier Decay and the Nature of Low-n to High-n Transfer Processes in Quasi-Two-Dimensional Layered Perovskites
Source: J Phys Chem C Nanomater Interfaces. 2023 Nov 27;127(48):23312–22. doi: 10.1021/acs.jpcc.3c05415 (PMC10711792; doi:10.1021/acs.jpcc.3c05415)
Supplement: Supplementary file 1 — jp3c05415_si_002.pdf [file jp3c05415_si_002.pdf]

SUPPLEMENTARY – Disentangling Hot Carrier Decay and the Nature of Low-n to High-n Transfer Processes in Quasi-Two-Dimensional Layered Perovskites

Lisanne M. Einhaus,[†] Xiao Zhang,[‡] Kaijian Zhu,[†] Jeroen P. Korterik,[¶] Robert Molenaar,[§] Sven H.C. Askes,[‖] Guido Mul,[†] Johan E. ten Elshof,[‡] and Annemarie Huijser*,[†]

†*PhotoCatalytic Synthesis Group, MESA+ Institute for Nanotechnology, University of Twente, 7500 AE, Enschede, the Netherlands*

‡*Inorganic Materials Science Group, MESA+ Institute for Nanotechnology, University of Twente, 7500 AE, Enschede, the Netherlands*

¶*Optical Sciences Group, MESA+ Institute for Nanotechnology, University of Twente, 7500 AE, Enschede, the Netherlands*

§*NanoBioPhysics Group, MESA+ Institute for Nanotechnology, University of Twente, 7500 AE, Enschede, the Netherlands*

‖*Department of Physics and Astronomy, Vrije Universiteit Amsterdam, De Boelelaan 1081, 1081 HV Amsterdam, the Netherlands*

E-mail: j.m.huijser@utwente.nl

Supplementary Material

X-ray diffraction (XRD)

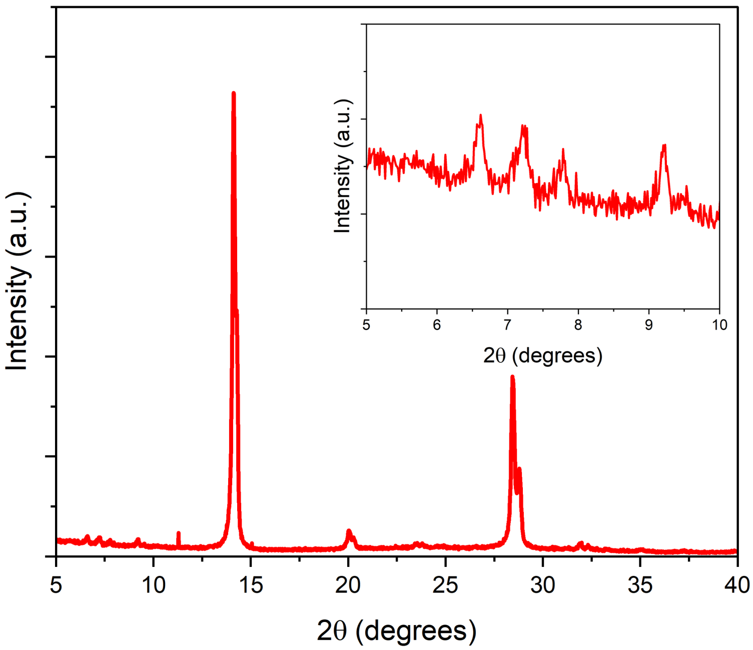

Figure S1: XRD pattern of $(PDMA)(MA)_{(n-1)}Pb_nI_{(3n+1)}$ ($\langle n \rangle$=5) perovskite film.

Figure S1 displays the X-ray diffraction (XRD) pattern of the perovskite film, $(PDMA)(MA)_{(n-1)}Pb_nI_{(3n+1)}$ ($\langle n \rangle$=5). The double XRD peaks at around 14.1° and 14.3° correspond to (002) and (110) lattice planes of the tetragonal $MAPbI_3$ phase. Similarly, the double peaks around 28.5° and 28.8° correspond to the (004) and (220) planes. The lattice reflection at 20.1° corresponds to a (200) phase. The low angle lattice reflections between 6° and 10° correspond to lower-n phases.

Scanning Electron Microscopy (SEM)

Figure S2 exhibits scanning electron microscopy (SEM) images of the $(PDMA)(MA)_{(n-1)}Pb_nI_{(3n+1)}$ ($\langle n \rangle$=5) perovskite thin film.

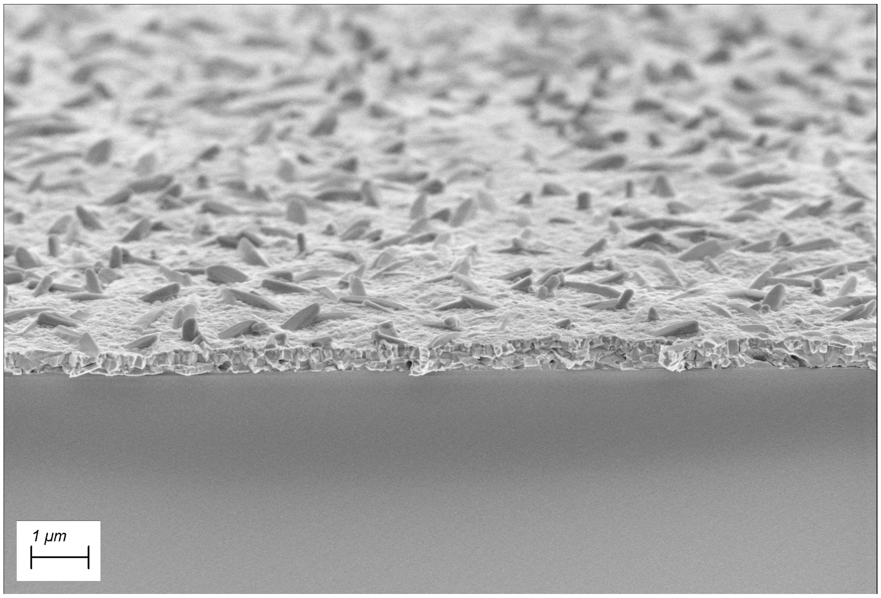

(a)

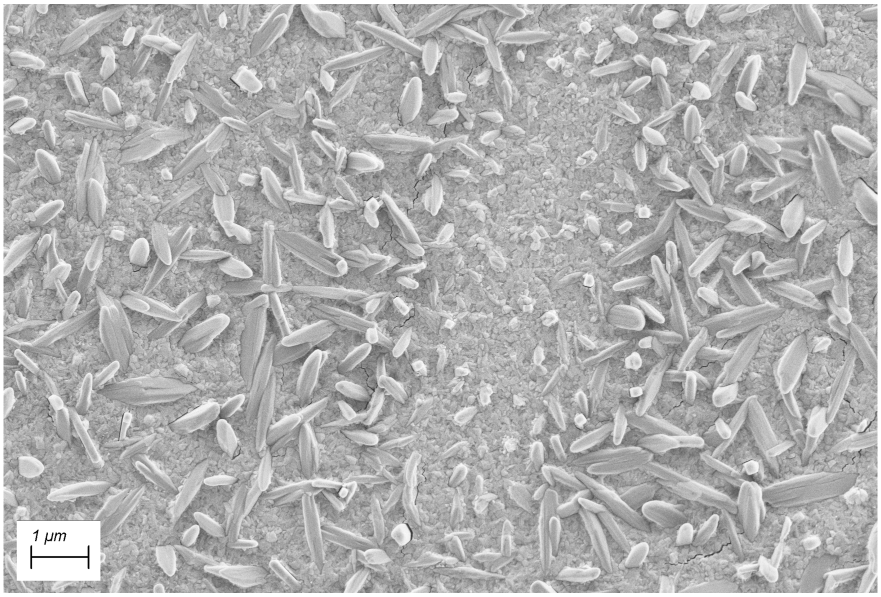

(b)

Figure S2: a) Cross-sectional and b) plan-view SEM images of $(PDMA)(MA)_{(n-1)}Pb_nI_{(3n+1)}$ ($\langle n \rangle$=5) perovskite thin film.

TRPL modeling

Figure S3 represents the species associated spectra obtained from target analysis on the TRPL data of $(PDMA)(MA)_{(n-1)}Pb_nI_{(3n+1)}$ ($\langle n \rangle$=5) perovskite thin film for photoexcitation at 532 nm. SAS1 represents the low-n phase, SAS2 the amorphous mixed n-phase, and SAS3 the high-n phase. Note that SAS1 was forced to zero for wavelengths >700nm, as the spectra would not offer a meaningful interpretation otherwise.

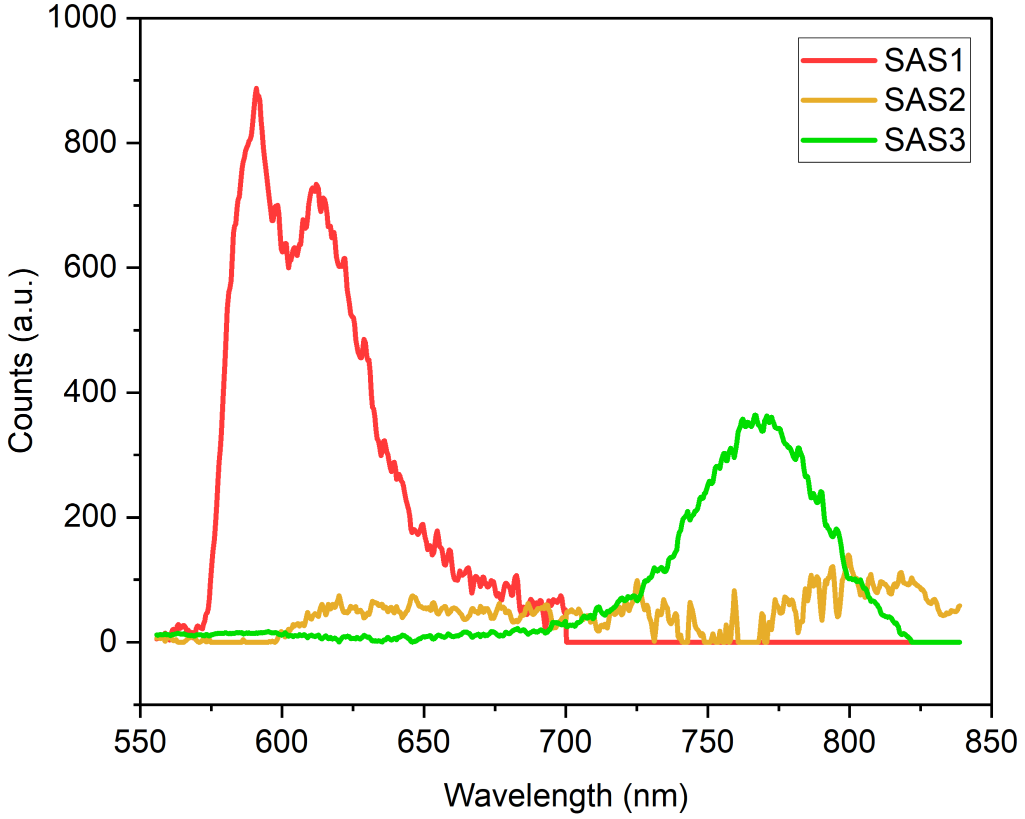

Figure S3: Species associated spectra obtained from target analysis on the TRPL data of $(PDMA)(MA)_{(n-1)}Pb_nI_{(3n+1)}$ ($\langle n \rangle$=5) perovskite thin film for photoexcitation at 532 nm.

TRPL front-side illumination

The TRPL spectra after front-side illumination (Figure S4) recorded at various times after photoexcitation at 532 nm show only one photoluminescence feature centered around 769 nm, analogous to the steady-state PL spectrum shown in Figure 1 of the main text, with the small deviations likely due to the different experimental setups used. This band represents the high-n phase. Note that the low-n bands are not resolved by illumination from this side.

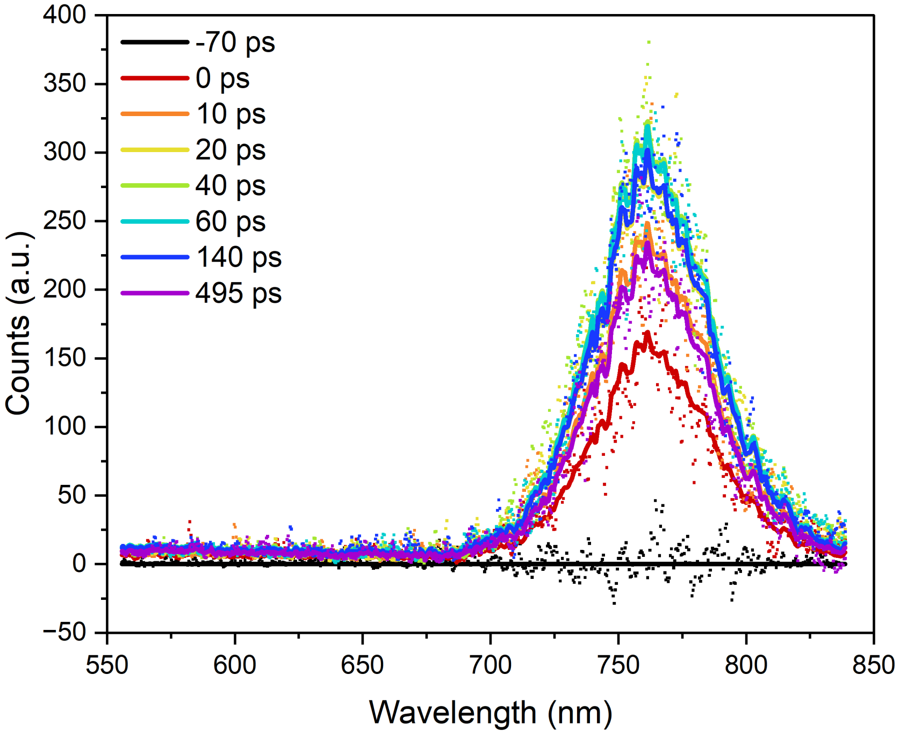

Figure S4: Time-Resolved Photoluminescence (TRPL) spectra at various times after 532 nm excitation measured by streak camera detection of $(PDMA)(MA)_{(n-1)}Pb_nI_{(3n+1)}$ ($\langle n \rangle$=5) recorded using front-side illumination and measured in reflection mode. Note that the spectrum is cut-off by a long-pass 570 nm filter.

In order to compare the kinetic traces using front-side and back-side illumination, the kinetic trace at the center of the front-side emission band was added to the graph representing the kinetic traces using back-side illumination (Figure S5).

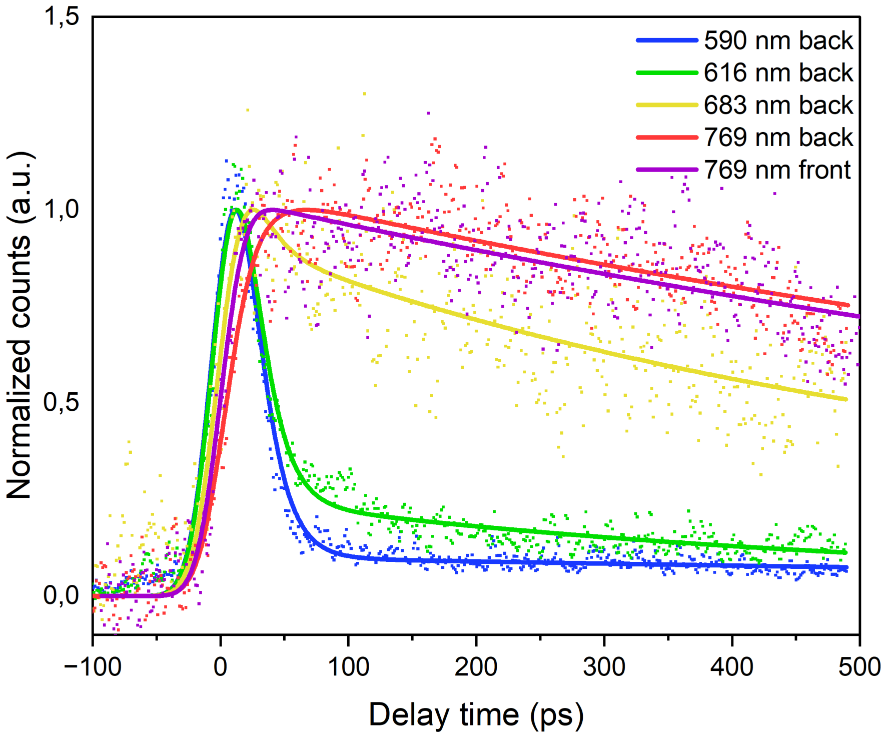

Figure S5: Time-Resolved Photoluminescence (TRPL) kinetic traces normalized to 1 at selected photoluminescence wavelengths measured by streak camera detection of $(PDMA)(MA)_{(n-1)}Pb_nI_{(3n+1)}$ ($\langle n \rangle$=5) recorded using back-side and front-side illumination and measured in reflection mode.

Extracting T_c from TA data

Preparation of the data

TA spectra modeled in Glotaran using target analysis as described in the main text were exported and used for this analysis, as the fits contain less noise compared to the raw data. As these spectra were defined in wavelength (nm) units, they needed to be converted to energy (eV) units first. Wavelength was converted to energy using $E = \frac{hc}{\lambda}$, where E is energy (eV), h is planck's constant (eV $\cdot$ s), c is the speed of light (m/s) and λ is wavelength (m). For conservation of energy after rescaling the units, the ΔA values were also scaled using a Jacobian transformation:[1] $f(E) = -f(\lambda)\frac{hc}{E^2}$.

General method: Fitting Maxwell-Boltzmann

The Fermi-Dirac distribution can be approximated by the Maxwell-Boltzmann distribution if $(E - E_f) >> k_b T_c$, where E_f is the quasi-Fermi energy, k_b the Boltzmann constant and T_c is the carrier temperature. A generally used method for determining carrier temperatures is to fit the high-energy tail of the normalized GSB to a Maxwell-Boltzmann distribution:[2,3]

$$\Delta A(E) \propto exp(-(E - E_f)/k_B T_c) \quad (1)$$

where $\Delta A(E)$ is the TA signal in the region of interest.

Fitting the Maxwell-Boltzmann distribution fails when the ΔA signal passes through zero, resulting in unrealistic carrier temperatures. Hence, the fitting region must be carefully chosen. This is further complicated by the fact that our TA data experience a redshift during the initial 1.5 ps, causing a shift in the energy level at which the zero crossing occurs.

Determining fitting region

After about 100 ps time delay, it is likely that the carriers have fully thermalized, as the high-n GSB redshift in the early time TA signal occurs <2 ps. Therefore, the fitting region

was determined by fitting a Gaussian function to some of the TA spectra $\geq$ 100 ps. A single Gaussian function is defined by:

$$f(x) = a1 * exp(-((x - b1)/c1)^2) \tag{2}$$

where $a1$ is the amplitude, $b1$ the centroid (location) and $c1$ is related to the peak width. The resulting coefficients and their 95% confidence bounds were a1 = -0.9843 (-1.021, -0.9475), b1 = 1.692 (1.689, 1.694) and c1 = 0.06789 (0.06496, 0.07082), and the fit is shown in Figure S6. The Gaussian fits the data well for energies between 1.6 eV and 1.8 eV. However, above ∼1.82 eV, the Gaussian is clipped to 0, while the TA data becomes positive indicating an ESA band. This indicates that the region above ∼1.82 eV should not be used for determining the carrier temperatures, as it is clear that multiple signals overlap. Therefore, 1.82 eV was set as the upper bound for fitting the Maxwell-Boltzmann equation.

The Maxwell-Boltzmann approximation works best when $(E - E_f) >> k_b T_c$. Therefore, the lower bound was set at as high energies as possible, such that at all delay times during the redshift there was a decent amount of datapoints to fit. In this case, 1.785 eV was set as the lower bound.

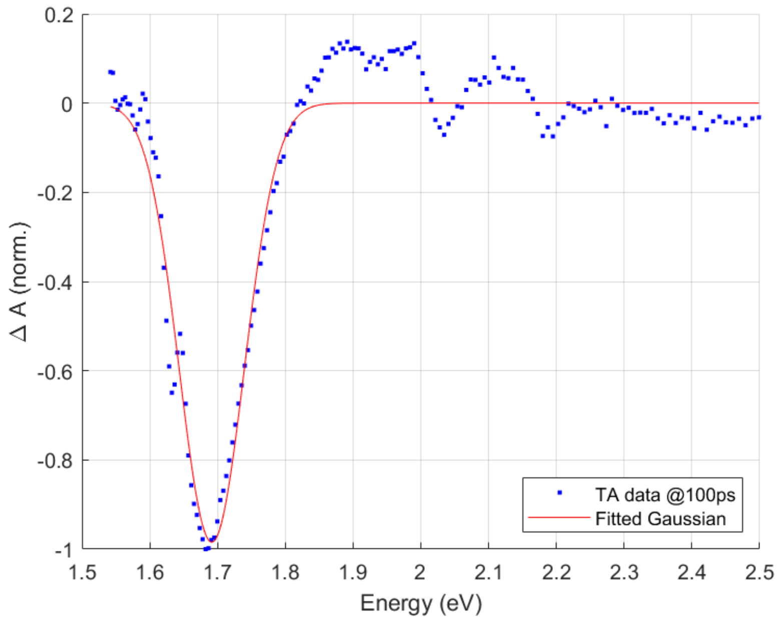

Figure S6: TA spectrum at 100 ps fitted with a single Gaussian function with a1 = -0.9843 (-1.021, -0.9475), b1 = 1.692 (1.689, 1.694) and c1 = 0.06789 (0.06496, 0.07082).

Fitting method

Fitting the Maxwell-Boltzmann function to the high-energy tail of the high-n GSB was performed in Matlab using the curve fitting add-on. The fit function was described by: fitfun = fittype(@(Te,A0,x) A0*exp(-(x-Ef)/(kb*Te))). The spectra were first normalized to the minimum value of the high-n GSB. Then, the high-energy side of the high-n GSB was fitted for the energy region between 1.785 eV and 1.82 eV. The value of E_f was set to be 1.7 eV.

Fitting results

The fitting was performed for the normalized TA spectra at several time delay times up to 5 ps and is shown as the bold lines in Figure S7. The fits are in good agreement with the data. Figure S8 shows the resulting carrier cooling curve, with the carrier temperatures extracted from the fits in Figure S7. The vertical error bars represent the 95% confidence interval of the fit. At early delay times, the carrier temperature reaches a maximum value of 1770 K

and then undergoes cooling towards approximately 240 K over a span of approximately 2 ps.

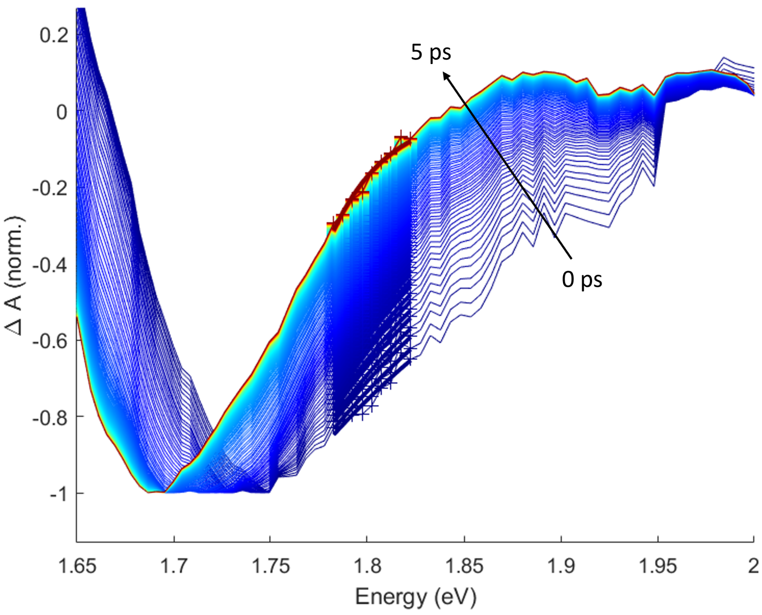

Figure S7: Normalized TA spectra of $(PDMA)(MA)_{(n-1)}Pb_nI_{(3n+1)}$ ($\langle n \rangle$=5) under 490 nm excitation, fitted with a Maxwell-Boltzmann function (bold).

Similar to previous observations,[3] we note that the carrier temperature curve slightly shifts depending on the chosen fitting region. Upon decreasing the lower energy boundary from 1.79 eV towards 1.77 eV, the final carrier temperature increases from approximately 280 K towards 400 K.

The fitting results provide a clear demonstration of hot carrier thermalization. However, the presence of the Gaussian shaped GSB centered at 1.7 eV and the presence of the ESA beyond 1.82 eV create a tight fitting window from which the hot carrier signal is difficult to retrieve. Therefore, we emphasize that the retrieved temperatures are a first order approximation of the absolute hot carrier temperatures.

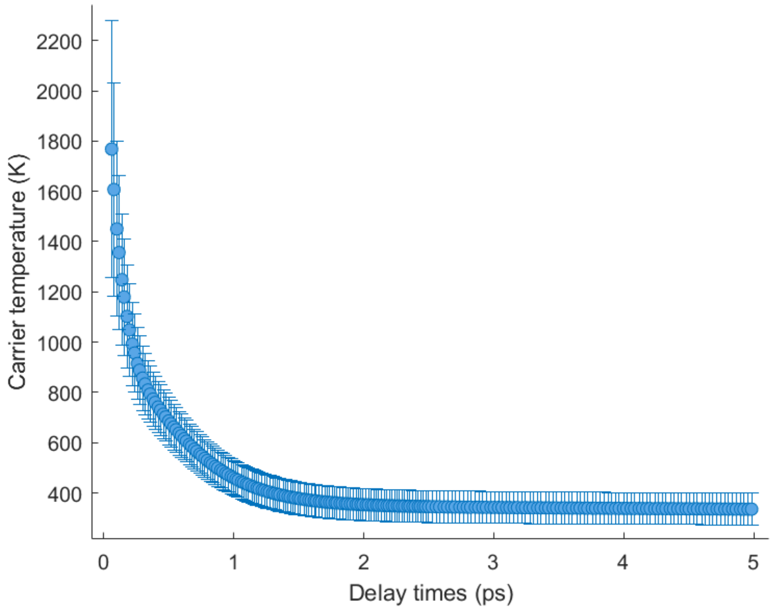

Figure S8: Carrier cooling curve for $(PDMA)(MA)_{(n-1)}Pb_nI_{(3n+1)}$ ($\langle n \rangle$=5) under 490 nm excitation. The carrier temperature T_c was extracted from the fitted normalized TA spectra.

References

(1) Mooney, J.; Kambhampati, P. Get the Basics Right: Jacobian Conversion of Wavelength and Energy Scales for Quantitative Analysis of Emission Spectra. *The Journal of Physical Chemistry Letters* **2013**, *4*, 3316–3318.

(2) Yang, Y.; Ostrowski, D. P.; France, R. M.; Zhu, K.; van de Lagemaat, J.; Luther, J. M.; Beard, M. C. Observation of a hot-phonon bottleneck in lead-iodide perovskites. *Nature Photonics* **2016**, *10*, 53–59.

(3) Lim, J. W. M.; Giovanni, D.; Righetto, M.; Feng, M.; Mhaisalkar, S. G.; Mathews, N.; Sum, T. C. Hot Carriers in Halide Perovskites: How Hot Truly? *The Journal of Physical Chemistry Letters* **2020**, *11*, 2743–2750, PMID: 32183508.
